# The Recruitment and Activation of Plasminogen by Bacteria—The Involvement in Chronic Infection Development

**DOI:** 10.3390/ijms241310436

**Published:** 2023-06-21

**Authors:** Dorota Satala, Aneta Bednarek, Andrzej Kozik, Maria Rapala-Kozik, Justyna Karkowska-Kuleta

**Affiliations:** 1 Department of Comparative Biochemistry and Bioanalytics, Faculty of Biochemistry, Biophysics and Biotechnology, Jagiellonian University, 30-387 Kraków, Poland; 2 Doctoral School of Exact and Natural Sciences, Jagiellonian University, 30-387 Kraków, Poland; 3 Department of Analytical Biochemistry, Faculty of Biochemistry, Biophysics and Biotechnology, Jagiellonian University, 30-387 Kraków, Poland

**Keywords:** plasminogen, plasmin, fibrinolysis, bacterial infection, enolase, periodontal disease

## Abstract

The development of infections caused by pathogenic bacteria is largely related to the specific properties of the bacterial cell surface and extracellular hydrolytic activity. Furthermore, a significant role of hijacking of host proteolytic cascades by pathogens during invasion should not be disregarded during consideration of the mechanisms of bacterial virulence. This is the key factor for the pathogen evasion of the host immune response, tissue damage, and pathogen invasiveness at secondary infection sites after initial penetration through tissue barriers. In this review, the mechanisms of bacterial impact on host plasminogen—the precursor of the important plasma serine proteinase, plasmin—are characterized, principally focusing on cell surface exposition of various proteins, responsible for binding of this host (pro)enzyme and its activators or inhibitors, as well as the fibrinolytic system activation tactics exploited by different bacterial species, not only pathogenic, but also selected harmless residents of the human microbiome. Additionally, the involvement of bacterial factors that modulate the process of plasminogen activation and fibrinolysis during periodontitis is also described, providing a remarkable example of a dual use of this host system in the development of chronic diseases.

## 1. An Introductory Overview of the Discussed Issues

The occurrence and further expansion of bacterial infections are closely related to the specific characteristics and activity of bacterial cell surfaces and extracellular hydrolysis capacity. For this reason, the important role of controlling the host proteolytic cascades by pathogens during invasion should not be overlooked when considering bacterial virulence mechanisms. This is the factor of crucial importance for the pathogen evasion of the host immune response, tissue damage, and pathogen invasiveness. One of such mechanisms is the bacterial impact on host plasminogen, the precursor of plasmin, an important plasma serine proteinase [1,2]. The numerous interconnections between the components of individual plasma proteolytic cascades, as well as regulatory mechanisms and interactions occurring at various levels, make the individual components of host systems, including plasminogen, a very convenient target to capture by microorganisms inhabiting host organisms to work to their advantage. For this purpose, the exposition of various proteins at the cell surface, responsible for binding to this host protein, might be exploited by pathogens, as by well as influencing plasmin activators or inhibitors. The involvement of bacterial factors that modulate the process of plasminogen activation and fibrinolysis provide a remarkable example of a dual use of this host system in the development of chronic diseases.

## 2. Plasminogen Functionality and Involvement in Physiological Processes

Plasminogen, a 92-kDa glycoprotein synthesized primarily in the liver and present in plasma at a concentration of 216 µg/mL (2.4 µM), is the precursor of serine proteinase—plasmin—and the key component of the fibrinolytic system, which is crucial in the process of the removal of fibrin clots formed after activation of the coagulation cascade [1,2]. This system consists additionally of plasminogen activators—tissue-type plasminogen activator (tPA) and urokinase-type plasminogen activator (uPA), which may be inhibited by members of the serpin family—plasminogen-activator inhibitors PAI-1 and PAI-2 [1]. Plasminogen and tPA bind to fibrin through exposed lysine residues, and this process facilitates zymogen activation to plasmin by both activators, even though uPA does not bind to fibrin, finally resulting in clot lysis to soluble fibrin degradation products (FDP). Furthermore, in the positive feedback mechanism, plasmin transforms the zymogen of plasminogen activator uPA into its active form [3,4]. Precise regulation of the system is crucial for maintaining hemostasis; therefore, it also includes inhibition of plasmin by its major inhibitors—serpin alpha2-antiplasmin (α2AP) and, additionally, non-serpin alpha2-macroglobulin (α2M)—through the formation of proteinase-inhibitor complexes. Plasmin can also be inhibited by the C1-inhibitor, which interacts with proteinase via the serpin mechanism; however, the C1-inhibitor is also a protein susceptible to degradation by plasmin when it is in a denatured polymeric state [5,6,7,8]. As long as plasmin and tPA are bound to fibrin during clot lysis, they are protected from binding by their main inhibitors—α2AP and PAI-1, respectively [1]. In addition, binding of fibrinolytic system proteases to the surface of human cells by a variety of different receptors also stimulates activation of the system and protects enzymes from inhibition [1,9]. Another aspect of system regulation is based on the removal of lysine residues exposed on fibrin by the action of thrombin-activatable fibrinolysis inhibitor (TAFI), similar to carboxypeptidase B, which prevents the binding of plasminogen to fibrin and its activation to plasmin [10].

Active plasmin exhibits a broad proteolytic activity with a preferential cleavage site after lysine or arginine residues, being active on a wide range of diverse proteins in the organism and thereby involved in different processes, from the canonical function in fibrin clot lysis, to ovulation, embryonic development, tissue remodeling, vascular remodeling, inflammation, and tumor metastasis. The substrates of this protease include von Willebrand factor, and extracellular matrix (ECM) proteins—laminin, fibronectin, and thrombospondin; plasmin may also activate matrix metalloproteinases, facilitating further degradation of ECM proteins. Furthermore, plasmin modulates the activity of coagulation factors V and IX and regulates complement activity via binding of factors C3 and C5, followed by the generation of chemotactic C3a and C5a activation fragments. Nevertheless, further degradation and inactivation of C3 and C5 have also been reported in other studies [1,11,12].

Plasmin action is known to be associated with the activation of immune cells after binding to certain cellular receptors, and this phenomenon is related to the production of inflammatory mediators and the propagation of inflammation, as plasmin also plays the role of an effective chemoattractant for immune cells [13]. One example is the involvement of plasmin in the stimulation of macrophages via the annexin A2 heterotetrametric complex receptor, with subsequent stimulation of the Janus kinase JAK1/TYK2 signaling cascade and the production of pro-inflammatory cytokines—tumor necrosis factor-alpha (TNF-α) and interleukin 6 (IL-6) [14]. Furthermore, several other receptor molecules responsible for plasmin binding have been identified so far for various cell types [13]. Considering also the initiation of the process of fibrin clot formation, during the proteolytic processing of fibrinogen by another important serum proteinase, thrombin, the pro-inflammatory fibrinopeptides A and B (Aα1–16 and Bβ1–14) are produced, and the latter is described as a significant chemoattractant for neutrophils, monocytes, and macrophages [15,16]. In addition, during the degradation of fibrin clots by plasmin, pro-inflammatory peptides and FDP are generated, i.e., D-dimers, D fragments, and E fragments, of which mainly the former are indicated as factors triggering the release of different cytokines from monocytes/macrophages, including interleukin 1α (IL-1α), interleukin 1β (IL-1β), and IL-6, and stimulating production of uPA, tissue factor (TF), and PAI-2 [17,18]. During fibrin hydrolysis by plasmin, other fibrin fragments are also produced, such as Bβ15-42 peptide and α-chain fragments, and, in contrast, the former exhibits immunosuppressive potential [16,19].

The fibrinolysis system is substantially inseparable from the process of activation of the coagulation cascade and the kinin generation system, and is tightly connected with the complement system, thus participating in defense mechanisms [20]. Such diverse activities associated with plasminogen and its active form—plasmin—make the role of this protein particularly important, not only in the processes related to hemostasis and tissue rearrangement, but also in the regulation of the inflammatory state, when it contributes to the development of acute inflammation, and may also be involved in the occurrence of chronic inflammatory conditions.

## 3. The Engagement of Plasminogen and Plasmin in Infections—Benefits and Drawbacks of Fibrinolytic System Activation by Pathogens

Bacterial microorganisms exploit several types of different interactions with the human host, ranging from existing as part of the physiological microflora required for the proper functioning of the human organism to being important etiological factors of various types of infections. The occurrence of disease caused by pathogenic bacteria results from a number of factors and phenomena, including not only the characteristics and activities of a particular pathogen, but also the efficiency of the host defense systems, as the development of infection usually depends on an imbalance between host immunity and microbial virulence, related to the pathogen’s ability to deregulate the processes that maintain homeostasis in the host organism and avoid defensive proteins and immune cells. Accordingly, the ability of microorganisms to bind to their cell surface a variety of host molecules, including plasma proteins—albumin, transferrin, and components of the complement system and coagulation cascade—has been frequently described, just as the binding of ECM proteins—laminin, fibronectin, collagen, entactin, or vitronectin. Moreover, the secretion of hydrolytic enzymes by pathogens during the invasion allows for the degradation of soluble host proteins in the bloodstream or proteins located within host tissues, and also for the hydrolysis of substrates bound and accumulated on the cell surface of the microorganisms. It is well documented that pathogenic bacteria may activate or inactivate different plasma proteolytic cascades involved in the maintenance of homeostasis and hemostasis in the host organism directly through the action of their proteinases or after capturing host components by surface-exposed bacterial receptors, affecting the operation of the entire system and leading to its facilitated activation, or to imprisoning host molecules, thus preventing their further actions [21,22,23,24].

**Figure 1:**
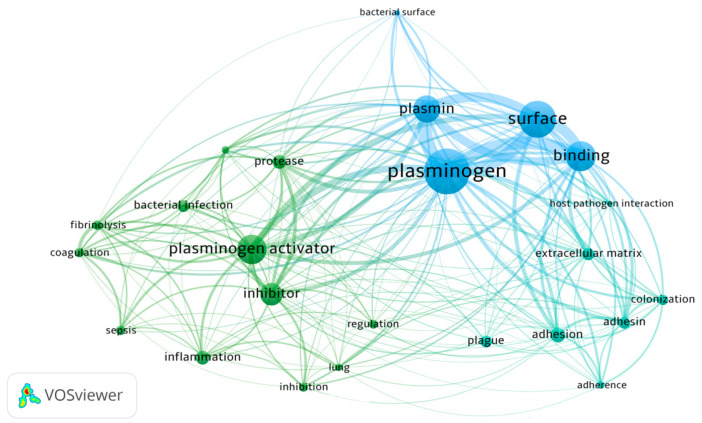
Network visualization of terms related to the relationship between plasminogen and bacterial infections noted in the scientific literature in the years 1992–2023 based on the Web of Science database (VOSviewer version 1.6.19).

As plasminogen is found in human plasma at a high concentration, and its active form, plasmin, demonstrates a broad proteolytic activity, the fibrinolytic system consisting additionally of plasminogen activators and inhibitors is a very useful target for disseminating pathogens, which can use many aspects of plasminogen functionality to their advantage. It should be mentioned that binding of plasminogen to the cell surface greatly enhances its hydrolysis by activators [25]; thus, the production and surface exposition of plasminogen-binding proteins may be one of the mechanisms beneficial to bacteria. The construction of a network of co-occurrence links between terms, carried out with VOSviewer and based on bibliographic data from the Web of Science Core Collection database from 1992 to 2023 with a search query for topics ‘plasminogen’ and ‘infection’ and ‘bacteria’, showed the noticeable presence of relations between these terms in the published scientific literature, referring to the activation of plasminogen to plasmin and regulation of the process of coagulation and fibrinolysis during host–pathogen interactions and manifestation of inflammatory conditions (Figure 1).

**Figure 2:**
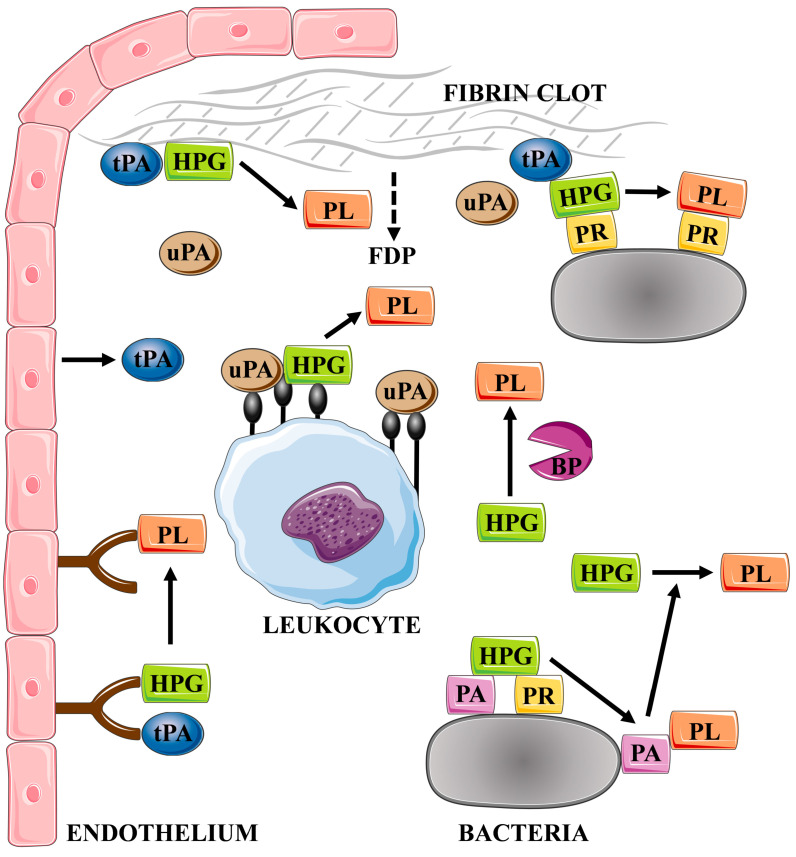
Mechanisms of the activation of the plasma fibrinolytic system by the host and by bacterial factors. The figure was partly generated using Servier Medical Art, provided by Servier, licensed under a Creative Commons Attribution 3.0 unported license. HPG—plasminogen, PL—plasmin, uPA—urokinase, tPA—tissue plasminogen activator, BP—bacterial proteinase, PA—plasminogen activator, PR—plasminogen receptor, and FDP—fibrin degradation products.

One of the primary mechanisms of the human host to combat bacterial invaders is to immobilize pathogen cells in a fibrin clot to prevent them from spreading in the bloodstream and constrain further development of infection, which is related both to the mechanical confinement of the cells and to the facilitation of the host immune response [23,26,27,28,29]. A key aspect of this host defense response is the association of the activity of host phagocytes with the presence of a fibrin network encapsulating pathogens [30]. This mechanism protects against the rapid expansion of extensive systemic infection, but does not prevent the development of a chronic disease, as microorganisms have acquired the ability to manipulate the clot formation and dissolution through various mechanisms (Figure 2) [31,32]. One example of how bacteria exploit the process of fibrin clot formation is the ability to assemble factors of the human coagulation cascade by *Escherichia coli* fibrous surface proteins—curli—facilitating activation of the coagulation pathway at the bacterial cell surface and generation of fibrinopeptides in the process regulated by *E. coli*-induced activation of the contact system comprised in the intrinsic pathway of the coagulation pathway. As a consequence, stimulation of immune cell migration and the induction of the proinflammatory cytokine MCP-1 at the inflammatory site is observed. This process on a local scale may support removal of pathogens by defense cells, and also facilitate progression of the disease related to the freer spread of bacteria released from the clot trap [33]. In addition, several fibrinogen- and fibrin-binding proteins have been identified for *Staphylococcus aureus*, including fibronectin-binding proteins A and B (FnBPA and FnBPB), clumping factors A and B (ClfA and ClfB), bone sialoprotein-binding protein (Bbp), *S. aureus* surface protein SdrE, and von Willebrand factor-binding protein (vWbp) [34,35]. For this bacterial species, it has also been demonstrated that the phenomenon of fibrinogen binding to the surface of pathogen cells can be important in the development of the host antibacterial response, but may also contribute to the formation of a mechanical barrier, protecting microbes against the activity of immune cells and favoring the formation of infectious lesions [34,36]. *Streptococcus pyogenes* M protein is also a well-known bacterial protein involved in fibrinogen binding [37,38,39]. Additional takeover of plasminogen/plasmin activity by wound-infecting streptococci allows the dissolution of the fibrin clot and, consequently, the dissemination of bacterial cells [40]. It has also been demonstrated that binding of fibrinogen- or fibrin-degradation products to the M protein of *S. pyogenes* protects bacterial cells from opsonization and phagocytosis [41].

The co-evolution of the human host and pathogenic bacteria resulted in a kind of “armaments race” and had the effect that the regulation of the interactions between fibrinogen/fibrin and plasminogen/plasmin actions is repetitively directed either to the benefit of the host and the activation of its defense mechanisms, or to the advantage of the bacterial pathogen [42]. Taking control of plasmin activity by bacteria gives them the opportunity to release from the fibrin trap, despite the fact that a fibrin clot may as well be a protective shield during the attack of immune cells, but, when the activity of the immune system is already extinguished, the surviving microbial cells can escape from the clot and give rise to a chronic infection. However, other studies have shown that active plasmin can prevent biofilm formation in the case of some bacterial species, including *Bordetella pertussis* and *Neisseria meningitidis* [43]. The host proteinase degrades important bacterial surface-exposed proteins—neisserial heparin-binding antigen (NHBA), neisserial IgA protease (IgAp), and B. pertussis filamentous hemagglutinin precursor (FhaB)—becoming part of the host defense mechanism against infection and colonization by harmful pathogens [43]. However, Arenas et al. [43] also reported that the biofilm produced by *S. aureus* was not susceptible to the anti-biofilm effect of proteinase, possibly because this bacterial pathogen has developed strategies to avoid the biofilm-inhibiting effect of plasmin. As previously noted by Kwiecinski et al. [44], in this species, the ability to form a biofilm in the presence of host proteins is strongly dependent on the level of expression of staphylokinase, which is a bacterial activator of plasminogen (described below). The less plasminogen activator *S. aureus* strain produces, the thicker biofilm it creates on the fibrin scaffold [44]. Thus, the process of bacterial fibrin-based biofilm formation and dispersion is dependent on the ability to adhere to fibrin, activate the fibrinolysis system, and maintain the balance between bacterial virulence factors and host defense mechanisms.

Apart from its main role in the degradation of fibrin clots, plasmin is also involved in the degradation of the ECM components, laminin and fibronectin, as well as activation of pro-collagenases, and this capability may also be exploited by pathogens to disseminate through host tissues after destruction of mechanical barriers [45]. In addition, plasmin proteolytic activity directed against host proteins can also be used to evade the immune system, including the degradation of important defense proteins such as opsonins or extracellular histones [46,47], or for hydrolysis of human cathelicidin—antimicrobial peptide LL-37 [48]. Hence, covering the surface of the bacteria with plasminogen and consequent activation of the system may facilitate the escape of bacteria from the initial site of infection and spreading in the host organism due to the ability to degrade ECM and basement membrane proteins, disintegrate tricellular tight junctions, and adhere and interact with endothelial and epithelial cells, as demonstrated so far for many different bacterial species and diverse infectious niches [42,49,50,51,52,53,54,55]. Interestingly, among the Lyme-disease-causing spirochetes, cp32 prophages might be involved in the horizontal transfer of genes encoding surface lipoproteins from the Erp family, whose representatives are involved in the binding of plasminogen and other host proteins [56,57,58]. This phenomenon sheds new light on the potential for dissemination of plasminogen-binding mechanisms in multi-species bacterial biofilm communities.

Interestingly, however, it is not only pathogenic bacteria that are involved in interactions with human plasminogen; microorganisms that are part of the physiological microflora and those with the characteristics of probiotic organisms are also able to affect the fibrinolysis system, as well as food functional bacteria such as particular *Lactobacillus* strains that can activate plasminogen to plasmin [59]. *L. plantarum*, commensal bacteria colonizing the human intestinal tract, can also bind plasminogen on the cell surface, leading to its activation and then the release of active plasmin into the surrounding; therefore, these bacteria do not exhibit prolonged surface proteolytic activity related to the host protein. Thus, they may support host defense mechanisms by competing for plasminogen binding with neighboring pathogens, whose cells become rather loaded with active plasmin used for further degradation and dissemination [60]. Additionally, for *L. crispatus*, extracellular release of some plasminogen-binding proteins, including enolase and glyceraldehyde-3-phosphate dehydrogenase, has been demonstrated, followed by enhanced activation of zymogen to plasmin in the presence of tPA and uPA; therefore, it may also constitute a particular form of a competition with pathogens in a co-colonized environment [61]. Furthermore, another probiotic strain, *L. reuteri*, demonstrated an effect on the downregulation of uPA gene expression [62], which indicates the existence of various mechanisms of interaction of harmless bacteria with the human fibrinolytic system.

## 4. Virulence Factors and Microbial Mechanisms Involved in the Hijacking of Fibrinolytic System by Pathogenic Bacteria

The degree of virulence of a particular pathogen is determined by properties such as the adhesion capability, efficiency of the production of toxins and hydrolytic enzymes, cell multiplication rate, flexible adaptation to environmental conditions, and evasion of host defense mechanisms, which are cooperatively related to the pathogen’s ability to colonize and destroy host tissues successfully [63,64,65]. With regard to the regulation of the fibrinolysis system and the effect on plasminogen, the important roles of secretory bacterial proteases, surface-exposed adhesive proteins, and plasminogen activators have been mainly specified.

Proteinases secreted by bacteria during infection are used not only to obtain nutrients from the environment, but also play a significant role in tissue colonization, microbial spreading in the host organism, and deregulation of host defense and immune processes [64]. These proteinases degrade immunoglobulins and complement proteins, facilitating the development of infection. They can also activate the host zymogens: plasminogen, procollagenase (matrix prometalloproteinase), and coagulation system proenzymes. Many bacterial proteolytic enzymes are not only insensitive to plasma proteinase inhibitors, such as serpins, but are also capable of their inactivation [63,66,67]. In general, any pathogen that extracellularly produces proteinases capable of hydrolyzing the Arg-X peptide bond can activate the complement, blood coagulation, and kinin generation cascades [64,68]. Bacteria produce proteinases belonging to all major classes, i.e., aspartyl, serine, cysteine, and metalloproteinases [64]. Some proteinases are not secreted into the environment, but are bound to the cell membrane through additional domains and are responsible for proteolytic activity on the surface of the bacterial cell [69]. One example is the outer membrane protease Pla from the omptin family of serine proteases, which is produced by the plague bacterium *Yersinia pestis*, capable of activating plasminogen to plasmin and inactivating the major plasmin inhibitor—α2AP, as well as plasminogen activator inhibitor PAI-1 [70,71,72,73]. Other representatives of the omptin family of transmembrane endopeptidases in gram-negative bacteria have also been identified as capable of PAI-1 inactivation, including PgtE protease from *Salmonella enterica*, Epo from *Erwinia pyrifoliae*, and Kop from *Klebsiella pneumoniae* [74]. Several omptins may also activate zymogen of uPA to active urokinase, including the abovementioned Pla and PgtE, as well as OmpT and OmpP from *E. coli*, SopA from *Shigella flexneri*, and Leo from *Legionella pneumophila* [75].

Additionally, bacteria often exploit activators that, through non-proteolytic transformations, trigger the activation of host plasminogen to plasmin. Streptococci produce streptokinase—a secretory, 47-kDa single-chain protein—which can form a complex with plasminogen, which is capable of activating other plasminogen molecules to plasmin, thus facilitating clot dissolution [42,76]. Activation of plasminogen by streptokinase on the bacterial surface results in degradation of extracellular histones and loss of their antimicrobial and hemolytic activity [47]. Similarly, *S. aureus* also produces 15-kDa plasminogen activator—staphylokinase—leading to the formation of active plasmin, often displayed on the surface of the bacterial cells and thereby protected against inhibition by α2AP, enabling bacterial cells to disseminate and to evade opsonization by exploiting the proteolytic properties of surface-bound plasmin for degradation of two main host opsonins—immunoglobulin G (IgG) and C3b fragment [46,77,78].

Surface proteins also contribute to the virulence of bacteria. The common feature of plasminogen receptors is that they are multifunctional molecules, often acting as adhesins to other proteins or host cells. They also have cell motility functions, influence the host immune response or, as in the case of moonlighting proteins, have important catalytic functions in the cytoplasm of the cell. Often, a single pathogen species expresses multiple receptors on its surface, which interact with plasminogen by cooperative action to increase plasmin activation, thereby influencing the success of the microbial spread in the host. Examples of plasminogen-binding proteins in pathogenic bacteria are presented in Table 1.

## 5. Enolase as the Best Studied Bacterial Plasminogen-Binding Protein—Structural Basis of the Interactions

Despite numerous reports on the interaction of bacterial surface proteins with plasminogen, only a few studies describe the structural basis of this interaction. The best-characterized example so far is enolase—a cytoplasmic enzyme classically involved in the glycolytic pathway. With the example of *S. aureus*, it was shown that the interaction between bacterial enolase and plasminogen plays a role in the microbial adherence to host cells, activates plasminogen with the participation of plasminogen activators, and also prevents α2-antiplasmin-mediated inhibition of plasmin [106].

Enolase is a highly conserved protein that shows more than 40% sequence identity in all species. It exists as a dimer or octamer, with each subunit consisting of a smaller N-terminal domain and a larger C-terminal domain [107]. Previous studies using free L-lysine and the analogous zwitterionic ligand ϵ-aminocaproic acid (EACA) suggested that the main sites involved in plasminogen binding are lysine residues exposed on the surface of bacterial enolase [81,84,108,109,110,111,112,113]. The key role of the last two C-terminal lysine residues in *S. pyogenes*, *S. pneumoniae*, *S. aureus*, *Mycobacterium tuberculosis*, and *Aeromonas hydrophila* was confirmed in studies based on site-directed mutagenesis, where removal of critical lysine residues or replacement with leucine, alanine, or asparagine resulted in a significant reduction in plasminogen binding [106,108,109,114,115].

Further analysis of bacterial enolase structures based on sequence comparisons provided evidence for the significant role of the internal 9-amino acid motif in the interaction with plasminogen [82,106,115,116,117,118,119]. In the case of several species of pathogenic bacteria—*S. aureus*, *S. pyogenes*, *M. tuberculosis*, *A. hydrophila*, and *Mycoplasma hyopneumoniae*, but also for commensal *L. plantarum*—the role of the internal motif was experimentally confirmed, while showing that the C-terminal lysine residues had an additive effect on binding of plasminogen [60,109,115,116,119]. In detail, the use of synthetic peptides corresponding to the internal motif of the enolase of *S. aureus* and *A. hydrophila* competitively inhibited its interaction with plasminogen, with the additional mutation of the C-terminal lysine that improved this effect [106,115]. In contrast, the substitution of a lysine residue at positions 193 and 194 in *M. tuberculosis* enolase led to a significant decrease in plasminogen binding, while the additional substitution of a lysine residue at position 429 contributed to a further reduction in binding [119]. The use of N- or C-terminal truncated enolase mutants of *M. hyopneumoniae* for direct binding assays and competition assays showed a significant reduction in plasminogen binding, especially for the C-terminal fragment, indicating the essential role of the C-terminal lysine residues in this interaction; while, for the N-terminus, a more important role in the activation step of plasminogen was suggested [120]. The remaining binding activity for the N- and C-terminal mutants was attributed to an internal enolase motif, which was previously shown to be partially located on the surface of the crystal structure of *M. hyopneumoniae* enolase [121]; however, this has not been confirmed experimentally [120]. Site-directed mutagenesis of *S. pyogenes* enolase showed that both the substitution of lysine residues in the internal motif at positions 252 and 255, and those present at the C-terminus at positions 434 and 435, resulted in a significant decrease in plasminogen binding [108,109]. The analysis performed for the model of octameric enolase demonstrated that the C-terminal lysine residues are located near the internal lysine residues, suggesting a common concerted function in plasminogen binding [109]. Thus, it is somewhat surprising that, recently, the involvement of C-terminal lysines and lysines present in the internal motif of *S. pyogenes* enolase in the binding of the solution-phase plasminogen has not been confirmed with site-directed mutagenesis [122]. Interestingly, as in previous in vitro studies [108,109], binding was observed when enolase adhered to different surfaces; however, this interaction was independent of C-terminal lysines [122]. Single-particle cryo-electron microscopy has shown that, for the closed octamer of *S. pyogenes* enolase, which is required for functional plasminogen binding, the C-terminal lysine residues are present in the minor interface between two monomers and are not available as plasminogen binding sites. According to the authors, the reasons for the observed differences are subtle changes in the enolase octameric conformation, which vary depending on the type of surface and result in a change in neoepitope exposure to plasminogen binding [122]. In the case of commensal *L. plantarum* enolase, which has only one lysine residue at the C-terminus of the protein, a single substitution of lysine at positions 259 and 442 has been reported to reduce plasminogen binding effectively, while a double substitution completely abolishes the interaction [60].

Furthermore, some examples of enolases have been reported in which plasminogen binding occurs via an internal domain independent of C-terminal lysine residues [113,116]. Namely, experiments based on site-directed mutagenesis of enolase of the probiotic bacteria *B. lactis* BI07 showed that the replacement of positively charged lysine at positions 251 and 254 by leucin and negatively charged glutamic acid at position 252 by glycine resulted in a significant weakening of plasminogen binding, while additional removal of the C-terminal lysines did not affect this interaction [82]. Contrary, for enolase from the infectious species *S. pneumoniae*, the analogous substitution of two lysins and glutamic acid residues in the internal motif was shown to result in a reduction in plasminogen binding to 44%, while the mutant with the deletion of C-terminal lysine residues did not show additional significant changes in plasminogen binding activity [116,123]. This observation is explained by a detailed crystallographic analysis, which showed that, in the pneumococcal enolase octamer, the C-terminal lysines are located in the inaccessible groove between the dimers, while the internal binding motif (248–256) is exposed on the surface of the octamer [117].

In addition, two observations have been reported to date that describe the interaction of enolases lacking C-terminal lysines in their sequences [115,118]. As expected, molecular docking for *H. influenzae* enolase showed that the internal bacterial protein motif (251–262) is involved in the interaction with plasminogen [118]. However, in the case of *A. hydrophila* enolase, reduced plasminogen binding activity was observed when lysine residues near the C-terminus at positions 420 and 427 were replaced by leucine and asparagine, respectively [115].

**Figure 3:**
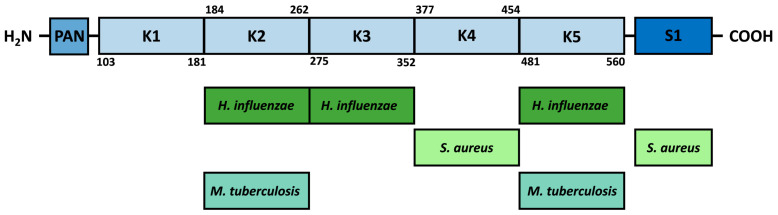
Proposed sites of interaction of bacterial enolases with individual plasminogen domains. PAN—plasminogen–apple–nematode domain, K1–K5—kringle domains, and S1—catalytic domain with serine protease activity.

The mature circulating form of plasminogen is known as Glu-plasminogen due to the presence of a glutamic acid residue at the N-terminus. In its structure, it contains a plasminogen–apple–nematode (PAN) domain located at the amino terminus [124], followed by five kringle domains (K1–K5), and then a catalytic domain with serine protease activity (S1) in the C-terminal region. Each of the kringle domains is highly stabilized by internal linkages, including three disulfide bridges, which makes them to be considered structurally and functionally independent. Kringle domains contain lysine-binding sites (LBS) that present shallow dipole-containing surfaces where a hydrophobic region of highly conserved aromatic residues separates opposite charges [125,126,127]. The kringle domains have been shown to interact via LBS with lysine residues present in many host receptors, such as fibrin or α2 antiplasmin, but also with free L-lysine and EACA; wherein the strength of lysine binding varies between domains. The strongest affinity was observed for the K1 domain, followed by K4, K5, and K2, and the weakest for K3 [125,128,129]. Despite the differences in binding strength observed, only a few studies of bacterial surface proteins concern a specific type of kringle domain; in the case of enolase, these are analyzed based on computational methods (Figure 3). The protein–protein docking for *M. tuberculosis* enolase indicated the K2 and K5 domains as fragments directly involved in the interaction with the internal enolase motif [119]. The interaction between proteins is stabilized by the formation of five hydrogen bonds: the first between enoGly272 and Ser194 within the K2 domain, then between enoLys193 and Arg504 within the K5 domain, with Arg504 simultaneously involved in bonds with enoSer190, which in turn forms two additional bonds with Arg471 and Lys468 presented within the K5 domain [119]. In contrast, the interaction of the internal *H. influenzae* enolase motif with plasminogen is conformationally stabilized by the formation of four hydrogen bonds: the first between enoTyr253 and Glu1 within the K2 domain, the second between enoTyr253 and Gly310 within the K3 domain, and the next two generated for the pairs enoLys255–Arg471 and enoGlu251–Lys468 within the K5 domain [118]. Moreover, the bioinformatics analysis performed for *S. aureus* showed that the interaction between the internal motif of enolase and the plasminogen is stabilized by four hydrogen bonds: two formed within the K4 domain between Asp362–enoTyr251 and Glu439–enoAsn253 and two formed within the S1 domain between Phe692–enoVal255 and Ala694–enoSer248 [106]. However, *S. aureus* enolase has been experimentally shown to interact with the five kringle domains with the same affinity as with full-length plasminogen; hence, it appears that binding within the K4 domain, rather than S1, is crucial for this interaction [106].

## 6. Modulation of the Fibrinolytic System by Bacteria in Periodontal Disease—Double-edged Sword in Chronic Infection

Periodontal disease is one of the most widespread chronic inflammatory-related diseases, manifested by the destruction of the soft tissues surrounding the teeth, followed by bone destruction, and consequently, loosening of teeth and their loss. Both in the acute phase of inflammation, manifested by bleeding, gingival crevicular fluid leakage, and swelling, as well as in the chronic phase of gingival disease, the plasminogen activation and fibrinolysis systems play a fundamental role. They mainly contribute to the process of wound healing, cell migration, tissue remodeling, and bone resorption and formation, as well as to the progression and resolution of inflammation [130,131,132]. In the etiology of periodontal disease, the key mechanisms of pathogenesis are related to the presence and activity of certain bacterial microorganisms in the lesion, which strongly contribute to the development and further control of inflammation and tissue destruction [133,134]. One of the important factors of microbial involvement is proteolytic activity in the infectious niche [68]. Both proteases secreted by bacterial periodontopathogens and deregulation of the host proteolytic activity by pathogens can contribute to the development of the disease [68,135,136,137,138]. *Porphyromonas gingivalis* and *Treponema denticola* proteinases—gingipains and dentilisin, respectively—may directly activate plasminogen to plasmin. Additionally, proteinases produced by several periodontal pathogens—*P. gingivalis*, *T. denticola*, *Prevotella intermedia*, and *Prevotella nigrescens*—have been identified as capable of degrading plasmin inhibitor α2AP [68,139]. The complex formed by *P. gingivalis* major cysteine proteinases—gingipains RgpA and Kgp—may activate zymogen of uPA [140]. Moreover, *P. gingivalis* Kgp is capable of degrading the plasminogen activator inhibitor PAI-1 in the periodontal tissues, resulting in intensified fibrinolysis, increased bleeding ensuring access to heme for bacteria, attenuated migration of the endothelial cells, and delayed wound healing, facilitating spreading of bacteria to other tissues [141].

Furthermore, it has been demonstrated that the *P. gingivalis* enzyme peptidylarginine deiminase (PPAD) is responsible, with the cooperation of gingipains, for the modification of at least six arginine residues in plasminogen to citrullines. However, citrullination did not affect either the process of activation of plasminogen to plasmin by gingipains, or the binding of human protein to the unmodified surface of *Candida albicans* yeast-like fungus, which may co-resident with *P. gingivalis* at the inflammatory site [142]. Although the binding of plasminogen to the cell surface may further facilitate its activation with plasmin, on the surface of *T. denticola* cells several proteinaceous receptors for plasminogen have been identified already, including proteins OppA, Msp, and FhbB. Similarly, plasminogen-binding activity was also assigned to the cell surface of *Fusobacterium nucleatum* subsp. *nucleatum* [139,143,144,145].

**Figure 4:**
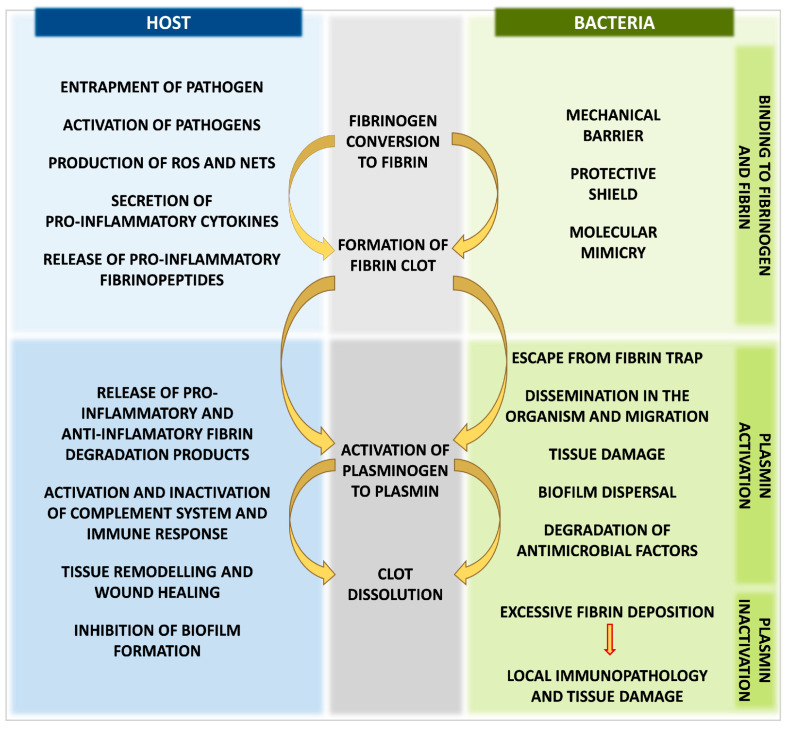
Advantages and disadvantages of fibrin clot formation and fibrinolysis system activation for human host and bacteria during host–pathogen interactions.

The other periodontopathogen—*Tannerella forsythia*—produces miropin, a protease inhibitor from the serpin superfamily with the ability to inhibit host plasmin effectively and specifically, which may protect these pathogens against degradation of bacterial proteins by plasmin and interfere with the role of plasmin in interactions with the activity of the immune system [146]. Furthermore, miropin can alter degradation of the fibrin deposits in periodontal tissues, being a product of increased thrombin activation and contributing to the pathogenesis of periodontal disease [146,147]. Excessive fibrin deposition and impaired fibrinolysis can lead to fibrin accumulation, persistent activation of neutrophils with the production of reactive oxygen species (ROS), neutrophil extracellular traps (NETs), and development of local immunopathology and periodontitis [148,149] (Figure 4).

Exaggerated plasmin activity associated with activation of the kinin production system and degradation of the ECM contributes to the development of inflammation, exudation, and destruction of periodontal connective tissue [13,150]. However, plasmin is also involved in the process of resolution of inflammation; therefore, the role of this enzyme in chronic infections such as periodontitis is multifaceted, as it contributes not only to acute and prolonged tissue damage, but also to the wound healing and bone repair [2,150,151,152,153]. It should also be remembered that subgingival plaque is composed of many different species of bacteria, so considering the influence of individual microorganisms on the plasminogen activation and fibrinolysis system may not fully reflect the complexity of interactions at the site of ongoing disease.

## 7. Conclusions

Close connection of the fibrinolysis system with the process of activation of the coagulation cascade, the kinin generation system, and the complement system draws attention to its essential role, not only in the physiological processes of wound healing and tissue remodeling, but also in the host defense mechanisms. Therefore, plasminogen and its active form, plasmin, are important factors involved in the regulation of the inflammatory state, both in acute inflammation and in chronic inflammatory conditions. The binding of human plasminogen to the cell surface of pathogens and its subsequent activation is a universal phenomenon and an important source of proteolytic activity, playing an essential role in the virulence process. Therefore, bacterial pathogens have developed a number of mechanisms that are involved in interactions with human protein, and one well-known example is the presence of a plasminogen-binding protein on the surface of bacterial cells, which is enolase. The involvement of various bacterial activities in the control of the fibrinolytic system may have two effects: it may lead to an exacerbation of the host inflammatory response, but it may also, in the longer term, cause the development of a latent, chronic infection. Therefore, a detailed understanding of the sophisticated interactions of bacteria with the host fibrinolytic system will contribute significantly to understanding the infection biology and may provide a starting point for alternative strategies to treat chronic inflammation.

## Figures and Tables

**Table 1:** Examples of bacterial proteins acting as receptors for plasminogen.

Surface Plasminogen-Binding Protein/Structure	Other Functions	Species of Bacteria	References
Fibronectin A and B binding proteins	Adhesion to host tissues mainly through their interactions with fibronectin	*S. aureus* *S. pneumoniae*	[79,80]
Enolase	Involved in glycolysis and adhesion to host proteins and cells	*S. pyogenes* *S. pneumoniae* *S. aureus* *Lactobacillus crispatus* *Lactobacillus johnsonii* *Bifidobacterium longum* *Bifidobacterium bifidum* *Bifidobacterium breve* *Bifidobacterium lactis* *Mycoplasma hyorhinis* *Borrelia burgdorferi*	[81,82,83,84]
Glyceraldehyde-3- phosphate dehydrogenase	Involved in glycolysis and adhesion to host proteins and cells	Group A streptococci *Bacillus anthracis* *Lactobacillus plantarum* *Clostridium perfringens* *E. coli*	[85,86,87,88,89,90]
Aspartase	Involved in nitrogen, alanine, and aspartate metabolism	*Haemophilus influenzae*	[91]
Phosphoglucomutase	Involved in gluconeogenesis	*Klebsiella pneumoniae*	[92]
Phosphoenolpyruvate carboxykinase	Involved in gluconeogenesis	*Klebsiella pneumoniae*	[92]
Triosephosphate isomerase	Involved in glycolysis	*S. aureus* *S. pneumoniae*	[93,94]
M-protein and M-like protein	Binding IgG and IgA, involved in the adhesion to epidermal keratinocytes, epithelial cell invasion, and microcolony formation	Group A streptococci	[40,95,96,97]
Fimbriae	Facilitating attachment to host tissues and promoting bacterial motility	*E. coli* *S. enteritidis*	[25,98,99]
Flagella	Facilitating attachment to host tissues and promoting bacterial motility	*E. coli*	[98,100]
OspA and OspC	Involved in host colonization	*Borrelia burgdorferi*	[101]
Neutrophil-activating protein	Involved in ROS production by neutrophil stimulation, and adhesion to endothelial cells	*Helicobacter pylori*	[102]
ClpC	Chaperone activity	*S. pneumoniae*	[103]
UvrC	Involved in DNA repair	*S. pneumoniae*	[103]
DnaK	Chaperone activity	*Bifidobacterium animalis* subsp. *lactis*	[104]
Lipoprotein	Involved in the integrity of the outer structure of the cell membrane	*E. coli*	[105]

## Data Availability

Not applicable.
